# Brain stimulation in zero gravity: transcranial magnetic stimulation (TMS) motor threshold decreases during zero gravity induced by parabolic flight

**DOI:** 10.1038/s41526-020-00116-6

**Published:** 2020-09-21

**Authors:** Bashar W. Badran, Kevin A. Caulfield, Claire Cox, James W. Lopez, Jeffrey J. Borckardt, William H. DeVries, Philipp Summers, Suzanne Kerns, Colleen A. Hanlon, Lisa M. McTeague, Mark S. George, Donna R. Roberts

**Affiliations:** 1 Brain Stimulation Division, Department of Psychiatry & Behavioral Sciences, Medical University of South Carolina, Charleston, SC 29425 USA; 2 Ralph H. Johnson VA Medical Center, Charleston, SC 29401 USA; 3 Department of Radiology, Medical University of South Carolina, Charleston, SC 29425 USA; 4 Department of Anesthesia and Perioperative Medicine, Medical University of South Carolina, Charleston, SC 29425 USA

**Keywords:** Neuroscience, Neurological disorders

## Abstract

We are just beginning to understand how spaceflight may impact brain function. As NASA proceeds with plans to send astronauts to the Moon and commercial space travel interest increases, it is critical to understand how the human brain and peripheral nervous system respond to zero gravity. Here, we developed and refined head-worn transcranial magnetic stimulation (TMS) systems capable of reliably and quickly determining the amount of electromagnetism each individual needs to detect electromyographic (EMG) threshold levels in the thumb (called the resting motor threshold (rMT)). We then collected rMTs in 10 healthy adult participants in the laboratory at baseline, and subsequently at three time points onboard an airplane: (T1) pre-flight at Earth gravity, (T2) during zero gravity periods induced by parabolic flight and (T3) post-flight at Earth gravity. Overall, the subjects required 12.6% less electromagnetism applied to the brain to cause thumb muscle activation during weightlessness compared to Earth gravity, suggesting neurophysiological changes occur during brief periods of zero gravity. We discuss several candidate explanations for this finding, including upward shift of the brain within the skull, acute increases in cortical excitability, changes in intracranial pressure, and diffuse spinal or neuromuscular system effects. All of these possible explanations warrant further study. In summary, we documented neurophysiological changes during brief episodes of zero gravity and thus highlighting the need for further studies of human brain function in altered gravity conditions to optimally prepare for prolonged microgravity exposure during spaceflight.

## Introduction

During spaceflight, astronauts onboard the International Space Station (ISS) experience unique environmental conditions including radiation exposure, altered atmospheric parameters, and microgravity. Understanding the effects of spaceflight on human health is important as more opportunities become available to send humans into space including the near-term reality of commercial suborbital and orbital flights^1,2^. Extensive research has documented that adaptive responses occur throughout the body during exposure to the spaceflight environment^3^. However, relatively little is known concerning the effects of microgravity on human brain function and health. Our group and others have demonstrated changes in brain structure on post-flight MRI in ISS astronauts and cosmonauts including a global upward positioning shift of the brain coupled with narrowing of the central sulcus and vertex cerebrospinal fluid spaces, and ventricular enlargement^4–6^. Although anatomical changes would be expected to result in changes in brain physiology, there have been virtually no studies of acute brain changes in weightlessness.

Transcranial magnetic stimulation (TMS) is a portable, noninvasive method for measuring cortical excitability by delivering electromagnetic pulses to the brain. When applied over the motor cortex, TMS depolarizes neurons in the corticospinal tract that result in an observable and quantifiable motor response in the muscles of the contralateral hand. The intensity of the TMS electromagnetic pulse required to activate the motor cortex depends on several factors, including cortical excitability and scalp to cortex distance. The minimum amount of electromagnetic power required to move the thumb is known as the resting motor threshold (rMT)^7^. The rMT is a standard measure of corticospinal excitability and is sensitive to various factors at the synaptic level (such as pharmacological agents)^8,9^ and morphological level (distance of TMS coil on the scalp to motor cortex)^10,11^. TMS can thus indirectly and noninvasively measure cortical excitability and is able to capture acute CNS changes, making it a potential tool to measure brain changes in microgravity.

We built custom, head-worn TMS systems that enable the exploration of TMS effects in zero gravity^12^. We then conducted a parabolic flight study in which we collected rMTs in 10 individuals before- during- and after parabolic flight to investigate whether TMS is feasible and safe to administer in zero gravity. Additionally, we aimed to determine whether the rMT changes as a function of gravity state. Our a priori hypothesis was that the amount of electromagnetism required for the rMT would be altered in zero gravity compared to Earth gravity due to acute changes in the central nervous system.

## Results

### Safety of TMS in zero gravity

There were no adverse events caused by the single pulse TMS administered in this experiment, irrespective of gravity state. Anti-nausea medications were not used in order to avoid confounding effects on cortical excitability. Three of the 10 participants experienced transient nausea with vomiting during flight. When it occurred, the nausea was after each participant’s rMT was acquired (parabola numbers: 22, 25, and 26, respectively) with no participant reports of nausea during their rMT recording. There were no other adverse consequences of rMT assessment during zero gravity.

### Motor threshold in zero gravity

**Fig. 1:**
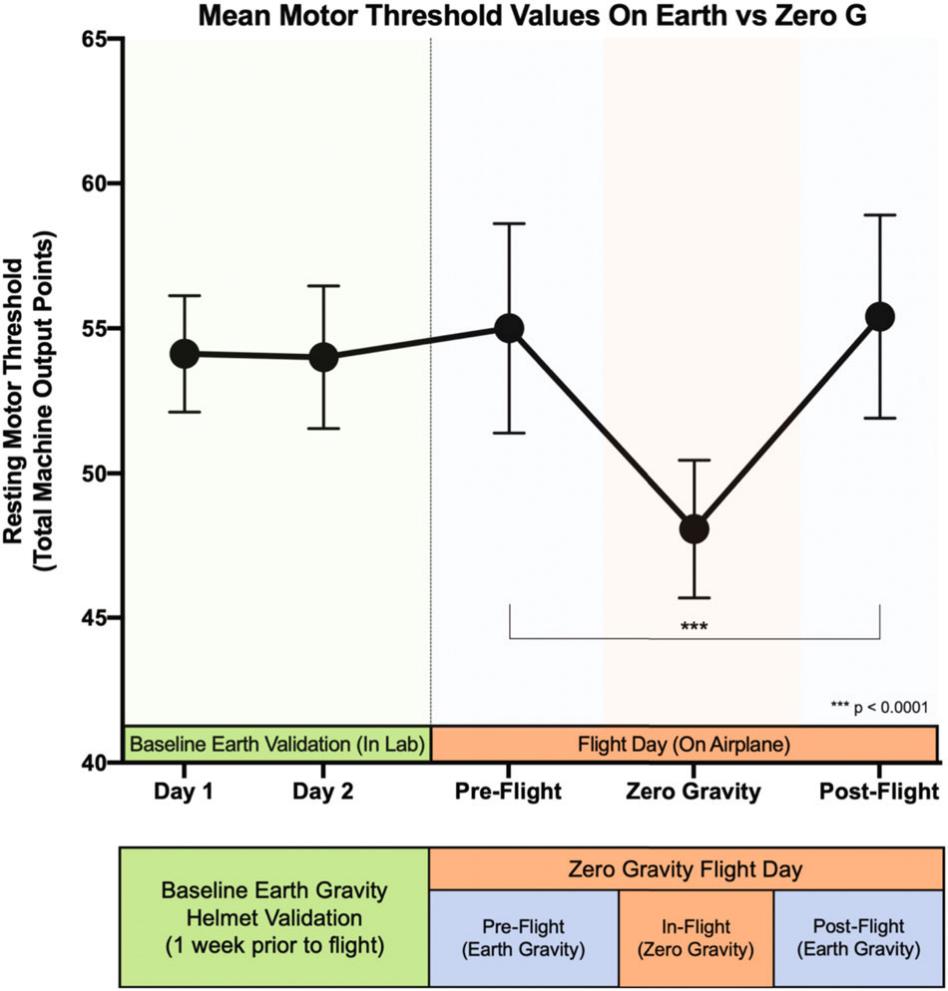
Motor threshold changes as a function of gravity state. (A) On Earth motor thresholds for the group (*n* = 10) remain stable at baseline and maintain the same average level through pre-flight measurements on the airplane. During Zero Gravity, a significant, 6.6 point reduction in motor threshold level was observed, which recovered post-flight (*p* < 0.0001).

We recorded the motor thresholds of 10 participants working in two teams of five people. Three to five rMTs were successfully acquired for each participant before (1 Gravity or G), during (0 G), and after (1 G) parabolic flight. The recordings during parabolic flight were measured during the zero gravity portions of each parabola, lasting approximately 20 s each. We found a significant effect of gravity state on TMS motor threshold (*F* (2,85.21) = 18.56, *p* < 0.0001) using a linear mixed-model, accounting for team (A or B), age, gender, subjective emotional arousal at the outset of motor threshold measurement, and rMT assessment number (1 to 5). Earth pre-flight (1 G) motor thresholds were a mean of 55.0 points (SE = 3.61). Parabolic flight (0 G) motor thresholds were a mean of 48.1 points (SE = 2.38). Upon return to Earth, the mean post-flight motor threshold was 55.4 points (SE = 3.50) (Fig. 1).

Overall, zero gravity motor thresholds were 6.6 (SE = 1.08) points lower than were Earth motor thresholds collapsed across pre- and post-flight timepoints (*t* (86.18) = 6.13, *p* < 0.0001). The immediately pre-flight motor thresholds were 6.6 points (SE = 1.11) higher than in 0 G (*t* (85.09) = 5.98, *p* < 0.0001), equating to a 12.6% reduction in motor threshold value. This reduction recovered immediately post-flight as the Earth post-flight (1 G) thresholds were 6.5 points (SE = 1.48) higher than in zero gravity (*t* (85.41) = 4.39, *p* < 0.0001), and roughly the same as before the flight. No significant difference was found between the pre- and post-flight Earth sessions (*F* (1,47.35) = 0.772, ns) and no significant effects were found for any of the other variables in the model: team, emotional arousal, age, gender, motor threshold or assessment number.

**Fig. 2:**
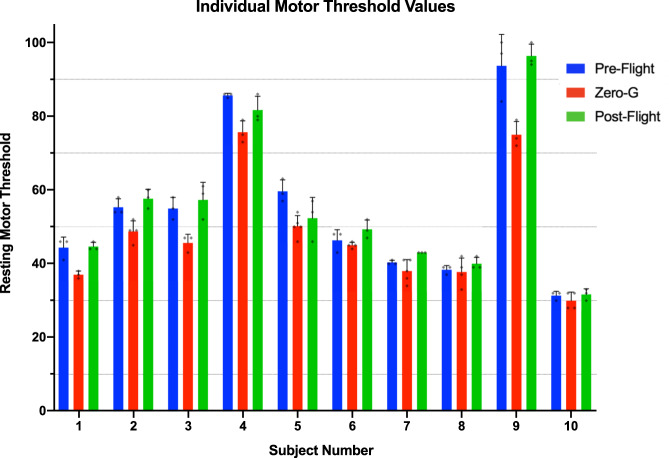
Individual resting motor threshold data across all measured time points demonstrating a reduction in motor threshold value for each individual during Zero-G periods compared to 1G onboard parabolic flight.

We further investigated the consistency and reliability of these overall group findings by looking at the individual effects of each of the 10 individuals on the flight. These findings are presented in Fig. 2 which demonstrate a consistent reduction in the resting motor threshold during zero-g time points compared to pre- and post flight. For all 10 individual fliers, the mean zero-g resting motor threshold value was lower than the pre- and post- flight motor threshold, suggesting this is a true biologic effect. Furthermore, the standard error for each of the individual measurements are similar at each time point.

### Subjective emotional arousal rating

We analyzed informal, self-reported subjective emotional arousal on a scale of 1 (lowest) to 10 (highest) at each motor threshold time point to determine whether emotional arousal may influence motor threshold levels. There was an overall main effect of time comparing pre- (mean 5.2, SEM 0.55), during- (mean 6.0, SEM 0.25), and post- (mean 3.7, SEM 0.63) flight emotional arousal (*F* (1.540, 13.86) = 9.92, *p* = 0.0035). This effect was driven by the post-flight reduction of emotional arousal, and post-hoc comparisons revealed no significant difference between pre- and during- flight emotional arousal.

We used a linear mixed model with unstructured covariance matrix to examine the effects of subjective emotional arousal ratings and found no significant effect of emotional arousal on the motor threshold values analyzed in this experiment (*F* (1,85.79) = 0.61, ns).

## Discussion

Using custom helmets and closed-loop, real-time EMG analysis software, we have demonstrated the feasibility of determining rMT during brief episodes of zero gravity induced by parabolic flight. Supporting our a priori hypothesis that the gravity state alters neurophysiology, we found that rMT levels were 6.6 points (or 12.6%) lower in zero gravity than they were pre- and post-flight in Earth gravity. These rMT changes were transient and did not persist after flight, and were not related to age, gender, or subjective emotional arousal at the time of data acquisition. Under normal conditions, the rMT is fairly consistent within an individual over time^13–16^ and is used as a standard measure in TMS treatment protocols to determine individual dosing. Therefore, the assessment of rMT in the zero gravity condition is an important baseline data point in understanding the response of the brain to acutely altered gravity and will facilitate investigators in designing TMS treatment protocols for use on future spaceflight missions.

The magnitude of the changes found are considered large when compared to pharmacologic methods of modulating cortical excitability such as the anticonvulsant medication lamotrigine^17^, with a similar range of effect size however opposite directional effect. However, there are many factors that can influence the magnitude of rMT changes, such as equipment (TMS machine and coil), the determination method of rMT (visual or EMG based), targeted muscle, participant characteristics (e.g. age, gender, etc.) and others^18^. Therefore, the mechanisms underlying the magnitude of change we observed in rMT during parabolic flight however are still unclear.

These findings suggest that physical movement of the brain within the skull during the alternating gravitational loads of parabolic flight may have been a contributing factor to our observed effects on rMT. TMS rMT varies widely between individuals, however, is extremely reliable within individual. Nearly 60% of the between individual variance is due to differences in the scalp to cortex distance^13^. As the scalp to cortex distance increases a greater amount of electromagnetism is required to induce cortical activation. Kozel et al. have suggested that within a narrow range, every 1 mm increase of scalp to cortex distance would result in a 2.9 point increase in TMS motor threshold^13^. If brain movement does occur acutely during parabolic flight, it could result in altered rMTs. Applying Kozel’s findings to our current study suggests that the 6.6-point reduction of rMT in zero gravity which we documented would have required an upward shift in the brain of approximately 2.3 mm. This magnitude of shift is plausible, as the average distance at the vertex between the surface of the brain and the endocast is, on average, 3–7 mm^19^.

The brain is a deformable tissue and is not rigidly fixed in place. During each cardiac cycle, the brain undergoes a deformation with the largest displacements occurring at the level of the brain stem. At the level of the cortex, peak displacements are approximately 0.1 mm^20,21^. Few studies have examined how much the brain may instantaneously shift under the altered directional gravity gradients experienced during normal daily position changes, and those studies have reported shifts on the order of the typical voxel size (1 mm). Mikkonen and Laakso^22^ reported an upward and backward shift of the brain in the supine position with the greatest shifts of up to 1.6 mm involving the parietal regions, although alignment errors were 0.4 ± 0.1 mm. Other investigators have also suggested that the brain may shift by approximately 1 mm when moving between the supine, lateral recumbent and prone positions, however these measurements were made by indirectly estimating brain movement based on estimating the thickness of the surrounding CSF^23,24^. It is unknown how much the brain may shift in position upon moving from supine to the upright position or during parabolic flight. Roberts and colleagues^5^ have previously demonstrated an upward shift of the brain in astronauts following months in space aboard the ISS, however, the chronic effects of exposure to weightlessness measured in 1 G would not be equivalent to the transient changes we describe here. However, we did not actually measure the brain’s position during parabolic flight so this is only one possible explanation for our results.

In addition to the physical movement of the brain, body position is known to acutely affect cerebral hemodynamics. For example, Alperin et al. has previously shown that compared with the supine position, CSF outflow through the foramen magnum while upright is decreased by 50%, cerebral blood flow is decreased by 12% and intracranial compliance is increased by 2.8 times^25^. Intracranial pressure (ICP) is known to change with changes in position as well as during parabolic flight. Lawley et al. found that ICP during parabolic flight is reduced in 0 G while lying in the supine position compared to 1 G^26^. Internal jugular venous pressure increases during parabolic flight compared with the supine position (23.9 ± 5.6 vs. 9.9 ± 5.1 mm Hg)^27^. A change in body position results in arterial baroreceptor stimulation which alters cortical activity and studies have shown that the supine position results in cortical inhibition^28–30^. It is possible that the dynamics of ICP or these physiological changes or both during parabolic flight altered cortical excitability and contributed to the decrease in rMT.

Interestingly, a prior, non-TMS parabolic flight experiment conducted in 2008 by Schneider and colleagues^31^ recorded resting electroencephalogram (EEG) activity in seven participants before, during, and after zero gravity which suggested that frontal lobe excitably might change in zero gravity. In contrast to our study, they found EEG suppression of frontal cortical excitability, rather than an increase during zero gravity. On the other hand, Chéron et al. found an increase in power of spontaneous 10-Hz oscillation on EEG in the parieto-occipital and sensorimotor areas in 5 cosmonauts during spaceflight^32^. It is not clear how these EEG measures relate to our TMS rMT findings. Cortical excitability of the motor system has also been investigated previously by Davey and colleagues in 2004^33^, who were perhaps the first to use TMS to explore corticospinal excitability in zero gravity. Davey administered TMS to the bilateral motor cortices to investigate motor changes in the lower extremities of three healthy individuals. They were only able to acquire valid data in one subject, however they found that this subject had transient increases in motor evoked potential amplitude recorded from the lower extremity in microgravity compared to Earth gravity consistent with our results.

An alternative explanation for our observed reduction in motor threshold during zero-G could be changes in the periphery. Since acquiring rMT requires activation of cortex, which secondarily activates the musculoskeletal system (measured from recordings on the anterior pollicis brevis of the contralateral hand), the rMT changes could be due to biomechanics of the periphery that are more sensitive to motor cortex outputs in zero gravity^7,8,34^. This could be due to neuromuscular junction changes, differences in propagation of efferent motor signal, or a combination of the two. Generally, a muscle that is partially activated pushing against gravity will have a lower rMT than will the same muscle when it is completely at rest^14^. We thus assumed that with respect to muscle activation affecting our measurements, in zero gravity there is less gravity to push against, so the muscle would be fully ‘at rest’ and the amount of electricity needed to cause changes in it would increase. Our findings of decreased electricity needed in zero gravity are in the opposite direction predicted by this reasoning. However, in our study, we did not explore the peripheral effects of zero gravity, and therefore it is not possible to fully control for potential gravitational effects on the musculoskeletal system. Future studies could include EMG recordings of several muscles throughout the flight to determine if there is a general muscle activating effect of entering zero gravity^8^.

### Limitations and future considerations

This study is a first attempt to investigate the use of TMS in zero gravity and has several limitations that should be considered for future parabolic flight experiments that utilize TMS as an investigational tool. First, we only collected motor threshold values, as determined by a parametric estimation via sequential testing. However other TMS electrophysiological values that may better elucidate brain changes and excitability such as MEP latency, paired pulse TMS, cortical silent period and input output curves were not collected. Future experiments assessing the effects of zero gravity on motor physiology could consider adding some of these additional measurements to further assess the effects observed in this study. Second, we only collected information during zero gravity periods and not during hyper-gravity, which limits the interpretation of the findings as to whether gravity state was the underlying cause of the changes rather than simply being on the flight. Third, we did not quantify the magnetic field (Tesla) emitted from the TMS machine, and although highly unlikely, since the systems used were intended for use in 1 G, the magnetic field strength produced by the TMS coil might conceivably have changed. Lastly, it is important to recognize that these preliminary findings are for brief periods of zero gravity and are difficult to translate to long-term spaceflight. Future studies could use TMS to investigate neurophysiological changes in subjects exposed to zero gravity for longer periods.

We have demonstrated that administering TMS in zero gravity aboard a parabolic flight in a team environment is safe and feasible, leading the way for future studies of brain physiology in zero gravity environments. We found that the rMT, a fundamental measurement in the application of TMS, significantly decreases in zero gravity induced by parabolic flight and restores to baseline levels post-flight. It is difficult to elucidate the underlying mechanism of our findings; however potential etiologies include upward brain shift, increased cortical excitability, changes in intracranial pressure, peripheral nervous system changes in the musculoskeletal system, or some combination of all of these. As TMS and other brain stimulation methods grow in their clinical utility, and likely need for use in space, further studies are needed to build on these findings. In addition, TMS rMT is an important tool to directly measure brain activity in zero gravity and more studies are needed to understand how the human brain adapts to zero gravity.

## Materials and methods

### Study overview

We recruited 10 healthy adults (5 men, mean age = 41.0, SD = 11.0) in this multi-visit TMS cortical excitability experiment conducted in simulated zero gravity (0 G) environment induced by parabolic flight (Zero Gravity Corporation, USA). Inclusion/exclusion criteria were as follows: Age between 25–61 years old, familiarity with TMS equipment, a baseline resting motor threshold lower than 90% of total machine output, no personal or familial history of seizures, no medications that would reduce seizure threshold, no metal implanted in the body above the level of the neck, no motion sickness on Earth. One of the 10 participants had prior zero gravity experienced. All others were unexperienced fliers who had limited to expert levels of TMS training and familiarity with the onboard TMS and MEP acquisition equipment. Nine out of 10 participants were right handed; handedness was not anticipated to impact rMT values as we used a within-subjects, repeated-measures design. All research conducted in this study complied with ethical regulations for work with human participants, and all subjects signed written informed consent approved by the MUSC IRB. Furthermore, the authors affirm that human research participants provided informed consent for publication of the images in all figures and in supplemental materials. We made custom TMS helmets for each subject using the methods described in Badran et al.^12^. This simple method allows for reliable administration of TMS in mobile or extreme environments during which TMS coil placement needs to be fixed outside of the laboratory. These helmets produce consistent and reliable TMS-induced motor evoked potentials in the contralateral abductor pollicis brevis (APB) muscle.

Participants attended 2 baseline visits followed by one parabolic flight (see the study timeline in Fig. 1). The baseline visits were conducted the week before the parabolic flight. Participants were divided into two teams of 5 individuals (Team A and B) and each team was assigned their own closed-loop TMS system. Both systems had identical hardware and software. The two teams were roughly equivalent in age (Team A—mean = 42.8 years, Team B—mean = 39.2) and gender (Team A—2 female, Team B—3 female). Both teams performed 5 closed-loop TMS motor thresholds on each other in a round robin fashion at three different time points: T1—in the airplane while stationary on the runway pre-flight, T2—in the airplane during 0 G, T3—in the airplane while stationary on the runway post-flight.

### Closed-loop TMS/EMG paradigm

TMS was administered using two identical closed-loop TMS/EMG systems. The TMS component used was the Magstim BiStim capacitor with a D70 remote coil and the EMG component used was the Cambridge Electronics EMG system (CED 1401, 1902), which uses electromyography (EMG) to measure the amplitude of the TMS motor evoked potential. The EMG recording (sensors placed on the right abductor pollicis bevis) is real-time analyzed using a companion Spike 2 software that uses prewritten software to determine whether muscle activation occurred (>150 μV) and changes the output of the TMS capacitor to the next probabilistic intensity based on parametric estimation via sequential testing (PEST) protocol^35,36^. We used a threshold of >150 μV at all timepoints in anticipation of increased latent electrical noise in the on-plane environment. Therefore, all laboratory earth data collection was also conducted at the 150uV threshold to maintain a controlled, unified threshold through all data acquisition points. At the three timepoints included in our statistical analysis (Pre-Flight 1 G, During Flight 0 G, Post-Flight 1 G), we used a maximum of 5 PEST steps using an interstimulus interval of 3.0–3.5 s^37,38^. We did not formally assess how the conventional lab-based TMS PEST protocol rMTs compared to the on-plane PEST protocol rMTs as the only rMTs included in the statistical analysis were acquired using the 5 PEST step method.

**Fig. 3:**
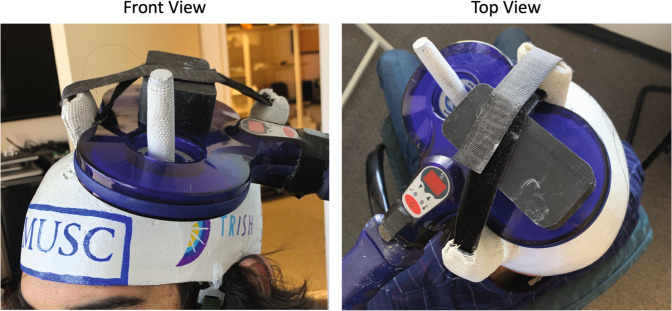
The TMS helmets used in in this experiment were custom casted to all participant’s head. We created 10 of these helmets, one for each flier. As visualized in this figure, helmets minimized any movement that could have been caused by weightlessness or shift in position.

All TMS was administered to the left motor cortex using custom, individualized, helmets designed to administer TMS in non-laboratory environments^12^ (Fig. 3) in a seated upright position. We did not collect roll, pitch, or yaw coordinate changes to track helmet stability as the helmets were custom cast to each individual’s head using fiberglass^12^. This tight fit greatly reduced the roll, pitch, and yaw of the helmet and had greater than 95% reliability of capturing accurate motor thresholds on two days. Furthermore, we minimized the risk of helmets floating in the superior direction during 0 G by attaching chin straps to each helmet and having the TMS administrator apply downward pressure to the TMS coil during the 0 G portions. All participants were additionally strapped into their seats with seatbelts. All motor thresholds were resting motor thresholds, with the participant’s right-hand resting palm-down on a foam pad with no muscles working against gravity as described in Badran et al.^39^. During the in-flight acquisitions, this foam pad was attached to the participants thigh to keep it from floating away and an elastic band was strapped to it to ensure the arm was in an identical position to all Earth motor thresholds.

We collected baseline rMT data to ensure repeatability and stability of the rMT of each participant. During each of the baseline visits, we collected five separate rMTs spread 1 min apart. The automated PEST system started at 50% maximum stimulation output (MSO) for each participant and used a series of incremental steps to determine the motor threshold.

### Parabolic flight TMS lab setup

**Fig. 4:**
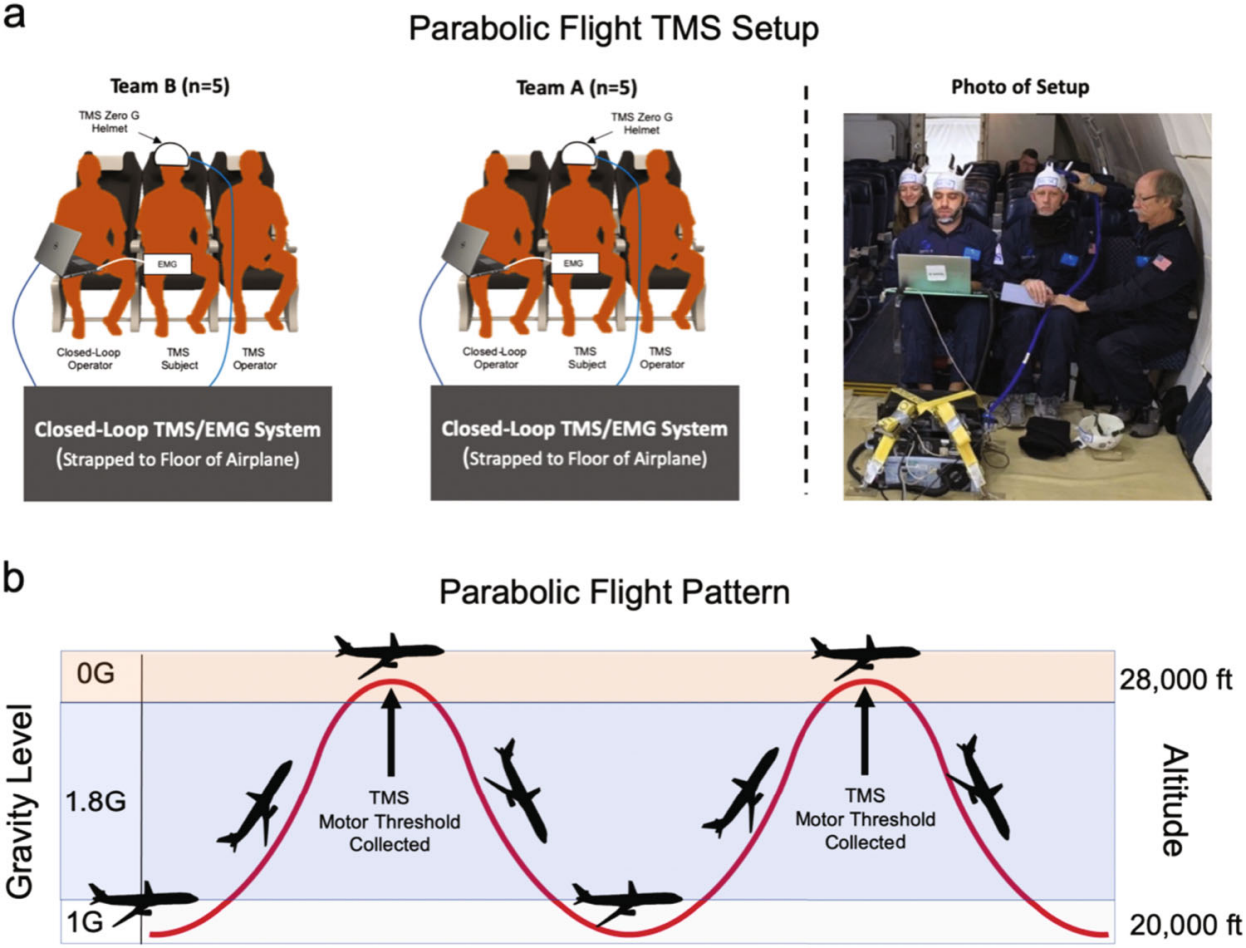
Overview of our TMS experiment in parabolic flight. **a** This diagram describes how the in-flight data collection was conducted. Each team had a computer operator, TMS operator, and a participant. Participants were rotated every 5 parabolas during level flight and received TMS using custom fabricated helmets that fix the TMS coil to the scalp. All TMS and EMG equipment was strapped to the floor of the airplane ahead of the computer operator and plugged into the airplane power circuit. **b** Parabolic flight simulates zero gravity by flying parabolas that alternate fliers between 1.8G and 0G. We administered TMS only during the 30 0G portions which each lasted approximately 20 seconds.

Parabolic flight was carried out in a modified Boeing 727 airplane prepared by Zero Gravity Corporation flying out of Sanford Airport in Sanford, Florida. We outfitted the plane with two mobile TMS laboratories capable of conducting closed-loop TMS with EMG recording and calculating an automated motor threshold in less than 20 s (Fig. 4a). The pattern of flight consisted of 30 parabolas, alternating between 1.8 G and 0 G as shown in Fig. 4b.

All on-plane rMT collections used the same modified rMT script for closed loop rMT determination. This modified script is a truncated version of the one used at the baseline the week before the flight, and rather than starting at 50%, was started at each individual’s average baseline determined rMT. This reduced the number of steps required to determine an rMT, shortening the time to <25 s, matching the limited time of microgravity induced during parabolic flight (<25 s). A **MOVIE** has been included that shows the research team A conducting the experiment in real time in footage from the flight.

For on-plane rMT collection while on the runway (pre- and post-flight), we collected 3 separate MTs for each participant spread 1 min apart. During each 25 s period of microgravity, we collected one rMT per team. We attempted to collect 5 rMT/subject. We collected a minimum of 3 MTs per participant (one rMT per parabola) and up to 5 MTs per participant depending on quality of acquisition. All on-plane MTs were conducted with the participant seated upright in a standard airplane seat. After 5 parabolas, participants rotated from receiving TMS to a different study-related task.

### Emotional arousal ratings

We collected subjective emotional arousal ratings to measure the impact of arousal on motor thresholds. This was a simple verbal rating scale prior to each flight day motor threshold (pre-, during- and post-flight). The participants were asked to rate their arousal on a scale from 1 (lowest) to 10 (highest).

### Statistical analysis

All rMTs were acquired on Spike2 recording software that recorded real-time EMG traces as well as the final motor threshold and were automatically stored on the computer after each motor threshold acquisition. After the flight, the computers containing the data were driven back to the laboratory for analysis. First—all EMG data was checked for quality control, ensuring no contamination from background noise or any false positives were indicated due to non-TMS recorded movement. No data was excluded during any of the Earth gravity rMT attempts (100 baseline attempts (5 per subject/visit), 30 pre-flight attempts (3 per subject), and 30 post-flight attempts (3 per subject)). During Zero Gravity, 10 of the 50 rMT attempts were rejected in-flight due to poor quality acquisition determined by the computer operator and secondarily confirmed digitally post-flight by one rater trained in Spike 2 software. Each participant had at least 3, and up to 5, clean zero gravity rMT acquisitions.

For determination of whether rMT changed with zero gravity, we used a linear mixed model with unstructured covariance matrix to examine the effects of session (Earth-pre-flight; zero-gravity; Earth-post-flight), controlling for participant age, participant sex, team (A or B), motor threshold assessment number and subjective emotional arousal ratings. Participant intercepts were entered into the model as random effects at level-1 (IBM SPSS 25).

We then used a linear mixed model with unstructured covariance matrix to determine whether motor threshold values were different during the Earth-pre-flight and Earth-post-flight sessions (again, controlling for participant age, participant sex, team (A or B), motor threshold assessment number and subjective emotional arousal ratings). Participant intercepts were entered into the model as random effects at level-1.

### Reporting summary

Further information on research design is available in the Nature Research Reporting Summary linked to this article.

## Supplementary information

In-Flight Video Footage of Experiment

Reporting Summary

## Data Availability

The data that support the findings of this study are available from the corresponding author upon reasonable request. The data are not publicly available due to containing information that could compromise research participant privacy. Please e-mail authors to request deidentified data and we will respond to any reasonable requests.
